# *trans*-Bis(methanol-κ*O*)bis­(quinoline-2-carboxyl­ato-κ^2^ *N*,*O*)manganese(II)

**DOI:** 10.1107/S1600536808031905

**Published:** 2008-10-11

**Authors:** Danuta Dobrzyńska, Lucjan B. Jerzykiewicz

**Affiliations:** a Faculty of Chemistry, Wrocław University of Technology, Wybrzeże Wyspiańskiego 27, 50-37 Wrocław, Poland; b Faculty of Chemistry, University of Wrocław, Joliot-Curie 14, 50-383 Wrocław, Poland

## Abstract

The title compound, [Mn(C_10_H_6_NO_2_)_2_(CH_4_O)_2_], was obtained unintentionally as the product of an attempt to synthesize a polynuclear carboxyl­ate bridged manganese(III/IV) complex, using methanol to reduce the permanganate ion. The mol­ecule is centrosymmetric; the pairs of equivalent ligands coordinate *trans* to each other in a distorted octa­hedral geometry. Intra­molecular C—H⋯O bonds lying in the equatorial plane stabilize the mol­ecule. In the crystal, mol­ecules are linked by O—H⋯O and C—H⋯O hydrogen bonds, creating a three-dimensional supra­molecular structure. π–π and C—H⋯π inter­actions are also observed. The dihedral angle and centroid-to-centroid distance between the pyridine ring (*A*) and the benzene ring (*B* ^i^) of a symmetrically related mol­ecule [symmetry code: (i) −1 − *x*, −*y*, −*z*] are 1.27 (11)° and 3.974 (2) Å, respectively. For the C—H⋯π inter­actions, the relevant distances and angles are: C⋯*Cg*[*A* ^ii^] = 3.643 (2) Å, H⋯*Cg*[*A* ^ii^] = 2.750 (2) Å and C—H⋯*Cg*[*A* ^ii^] = 155 (1)° [symmetry code: (ii) *x*, −1 + *y*, *z*].

## Related literature

For previously reported Mn^II^ complexes with the quinoline-2 carboxyl­ate ligand, see: Okabe &Koizumi (1997 ▶); Goher & Mautner (1993 ▶); Haendler (1996 ▶); Dobrzyńska & Jerzykiewicz (2004 ▶); Dobrzyńska *et al.* (2005 ▶, 2006 ▶).
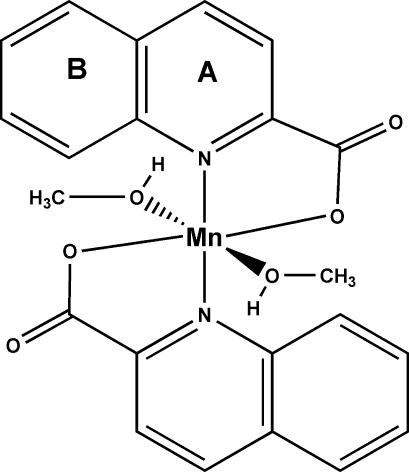


## Experimental

### 

#### Crystal data

[Mn(C_10_H_6_NO_2_)_2_(CH_4_O)_2_]
*M* *_r_* = 463.34
Monoclinic, 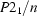

*a* = 10.596 (5) Å
*b* = 7.243 (3) Å
*c* = 13.534 (3) Å
β = 106.59 (4)°
*V* = 995.5 (7) Å^3^
*Z* = 2
Mo *K*α radiation
μ = 0.71 mm^−1^
*T* = 100 (1) K
0.43 × 0.12 × 0.09 mm

#### Data collection

Kuma KM-4 CCD κ-axis diffractometer
Absorption correction: analytical (*CrysAlis RED*; Oxford Diffraction, 2006 ▶) *T*_min_ = 0.873, *T*_max_ = 0.902
5405 measured reflections
1924 independent reflections
1475 reflections with *I* > 2σ(*I*)
*R*_int_ = 0.031

#### Refinement

*R*[*F* ^2^ > 2σ(*F* ^2^)] = 0.034
*wR*(*F* ^2^) = 0.089
*S* = 0.98
1924 reflections
146 parameters
H atoms treated by a mixture of independent and constrained refinement
Δρ_max_ = 0.37 e Å^−3^
Δρ_min_ = −0.31 e Å^−3^

### 

Data collection: *CrysAlis CCD* (Oxford Diffraction, 2006 ▶); cell refinement: *CrysAlis RED* (Oxford Diffraction, 2006 ▶); data reduction: *CrysAlis RED*; program(s) used to solve structure: *SHELXTL-NT* (Sheldrick, 2008 ▶); program(s) used to refine structure: *SHELXTL-NT*; molecular graphics: *SHELXTL-NT*; software used to prepare material for publication: *SHELXTL-NT*.

## Supplementary Material

Crystal structure: contains datablocks I, global. DOI: 10.1107/S1600536808031905/su2065sup1.cif

Structure factors: contains datablocks I. DOI: 10.1107/S1600536808031905/su2065Isup2.hkl

Additional supplementary materials:  crystallographic information; 3D view; checkCIF report

## Figures and Tables

**Table 1:** Hydrogen-bond geometry (Å, °)

*D*—H⋯*A*	*D*—H	H⋯*A*	*D*⋯*A*	*D*—H⋯*A*
O3—H3⋯O2^i^	0.80 (3)	1.83 (3)	2.623 (3)	172 (3)
C2—H2*A*⋯O1^ii^	0.93	2.58	3.411 (3)	148
C8—H8*A*⋯O1^iii^	0.93	2.36	3.241 (3)	158

Symmetry codes: (i) 
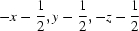
; (ii) 
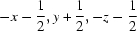
; (iii) 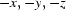.

**Fig. 1.**
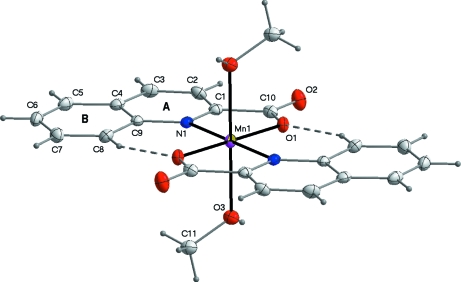
The molecular structure of compound (I), with atom labels and 50% probability displacement ellipsoids for non-H atoms.

**Fig. 2.**
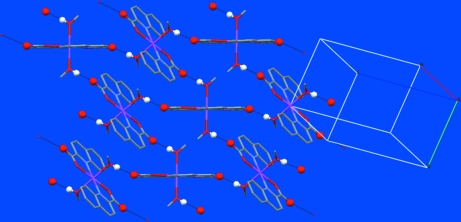
The crystal packing of compound (I), showing one layer of molecules connected by O—H···O and C—H···O hydrogen bonds (dashed lines). H and O atoms participating in O—H···O bonds are shown as balls.

## supplementary crystallographic information

### Comment

The quinoline-2-carboxylate (quin-2-c) ion is known as an effective chelator. A few Mn(II) complexes with the quin-2-c ion and different coligands have been reported previously (Okabe *et al.*, 1997, Goher *et al.*, 1993, Haendler, 1996, Dobrzyńska *et al.*, 2004, 2005, 2006). The title complex, (I), is centrosymmetric (Fig. 1). The quin-2-c ion coordinates in a typical O,*N* chelate mode. The pairs of the equivalent ligands lie *trans* to each other in a distorted octahedral geometry. The bite angle of the chelating ligand is 74.93 (7)° and falls in the range observed for other manganese(II) complexes with the quin-2-c ion (73.1° - 78.5°; see references quoted above). The intramolecular C—H···O bonds lying in the equatorial plane stabilize the molecule (Table 1).

In the crystal molecules are linked by O—H···O and C—H···O hydrogen bonds creating a three-dimensional supramolecular structure (see Table 1 and Fig. 2). π···π and C-H···π interactions are also observed. The dihedral angle and centroid-to-centroid distance between rings A [= N1,C1-C4,C9] and B^i^ [= (C4-C9)^i^; symmetry code (i) -1-x, -y,-z)] are 1.27° and 3.974 Å, respectively. For the C-H···π interactions the relevant distances and angles are: d(C11···Cg[A^ii^] = 3.643 Å, d(H11A···Cg[A^ii^]) = 2.750 Å with angle (C11-H11A···Cg[A^ii^] = 155° (symmetry code (ii) x, -1+y, z).

### Experimental

Compound (I) was obtained unintentionally as the product of an attempt to synthesize the polynuclear, carboxylate bridged manganese(III/IV) complex mixing a methanol solution of quinoline-2-carboxylic acid and potassium permanganate at room temperature.

### Refinement

The hydroxyl H-atom was located in a difference Fourier map and refined isotropically with the O-H distance restrained to 0.80 (3) Å. The C-bound H-atoms were included in calculated positions and treated as riding atoms: C-H = 0.93 - 0.96 Å with U_iso_(H) = 1.2 or 1.5U_eq_(parent C atom).

### Crystal data

**Table:**

[Mn(C_10_H_6_NO_2_)_2_(CH_4_O)_2_]	*F*(000) = 478
*M**_r_* = 463.34	*D*_x_ = 1.546 Mg m^−^^3^
Monoclinic, *P*2_1_/*n*	Mo *K*α radiation, λ = 0.71073 Å
Hall symbol: -P 2yn	Cell parameters from 3842 reflections
*a* = 10.596 (5) Å	θ = 3–26°
*b* = 7.243 (3) Å	µ = 0.71 mm^−^^1^
*c* = 13.534 (3) Å	*T* = 100 K
β = 106.59 (4)°	Block, yellow
*V* = 995.5 (7) Å^3^	0.43 × 0.12 × 0.09 mm
*Z* = 2	

### Data collection

**Table:**

Kuma KM-4-CCD κ-axis diffractometer	1924 independent reflections
Radiation source: fine-focus sealed tube	1475 reflections with *I* > 2σ(*I*)
graphite	*R*_int_ = 0.031
ω scans	θ_max_ = 26.0°, θ_min_ = 3.2°
Absorption correction: analytical (CrysAlis RED; Oxford Diffraction, 2006)	*h* = −13→12
*T*_min_ = 0.873, *T*_max_ = 0.902	*k* = −8→6
5405 measured reflections	*l* = −16→16

### Refinement

**Table:**

Refinement on *F*^2^	Primary atom site location: structure-invariant direct methods
Least-squares matrix: full	Secondary atom site location: difference Fourier map
*R*[*F*^2^ > 2σ(*F*^2^)] = 0.034	Hydrogen site location: inferred from neighbouring sites
*wR*(*F*^2^) = 0.089	H atoms treated by a mixture of independent and constrained refinement
*S* = 0.98	*w* = 1/[σ^2^(*F*_o_^2^) + (0.0573*P*)^2^] where *P* = (*F*_o_^2^ + 2*F*_c_^2^)/3
1924 reflections	(Δ/σ)_max_ < 0.001
146 parameters	Δρ_max_ = 0.37 e Å^−^^3^
0 restraints	Δρ_min_ = −0.31 e Å^−^^3^

### Special details

**Table:**

Geometry. All e.s.d.'s (except the e.s.d. in the dihedral angle between two l.s. planes) are estimated using the full covariance matrix. The cell e.s.d.'s are taken into account individually in the estimation of e.s.d.'s in distances, angles and torsion angles; correlations between e.s.d.'s in cell parameters are only used when they are defined by crystal symmetry. An approximate (isotropic) treatment of cell e.s.d.'s is used for estimating e.s.d.'s involving l.s. planes.
Refinement. Refinement of *F*^2^ against ALL reflections. The weighted *R*-factor *wR* and goodness of fit *S* are based on *F*^2^, conventional *R*-factors *R* are based on *F*, with *F* set to zero for negative *F*^2^. The threshold expression of *F*^2^ > σ(*F*^2^) is used only for calculating *R*-factors(gt) *etc*. and is not relevant to the choice of reflections for refinement. *R*-factors based on *F*^2^ are statistically about twice as large as those based on *F*, and *R*- factors based on ALL data will be even larger.

### Fractional atomic coordinates and isotropic or equivalent isotropic displacement parameters (Å^2^)

**Table:**

	*x*	*y*	*z*	*U*_iso_*/*U*_eq_	
Mn1	0.0000	0.0000	0.0000	0.01282 (16)	
O1	−0.06256 (14)	0.0841 (2)	−0.15489 (11)	0.0151 (3)	
O2	−0.21857 (15)	0.2157 (2)	−0.28166 (11)	0.0235 (4)	
O3	−0.08755 (15)	−0.2745 (2)	−0.04702 (12)	0.0179 (4)	
N1	−0.21067 (17)	0.1118 (2)	−0.02417 (13)	0.0126 (4)	
C1	−0.2606 (2)	0.1766 (3)	−0.11886 (16)	0.0137 (5)	
C2	−0.3844 (2)	0.2649 (3)	−0.15224 (16)	0.0166 (5)	
H2A	−0.4157	0.3101	−0.2191	0.020*	
C3	−0.4573 (2)	0.2825 (3)	−0.08415 (17)	0.0187 (5)	
H3A	−0.5391	0.3400	−0.1047	0.022*	
C4	−0.4091 (2)	0.2138 (3)	0.01703 (16)	0.0156 (5)	
C5	−0.4793 (2)	0.2244 (3)	0.09220 (17)	0.0184 (5)	
H5A	−0.5612	0.2818	0.0756	0.022*	
C6	−0.4280 (2)	0.1518 (3)	0.18819 (17)	0.0196 (5)	
H6A	−0.4751	0.1600	0.2364	0.024*	
C7	−0.3044 (2)	0.0644 (3)	0.21473 (17)	0.0184 (5)	
H7A	−0.2713	0.0136	0.2802	0.022*	
C8	−0.2318 (2)	0.0528 (3)	0.14573 (16)	0.0153 (5)	
H8A	−0.1494	−0.0031	0.1647	0.018*	
C9	−0.2833 (2)	0.1269 (3)	0.04511 (16)	0.0132 (5)	
C10	−0.1752 (2)	0.1571 (3)	−0.19219 (16)	0.0145 (5)	
C11	−0.1393 (2)	−0.3854 (3)	0.02034 (17)	0.0207 (5)	
H11A	−0.1730	−0.4990	−0.0136	0.031*	
H11B	−0.2090	−0.3196	0.0373	0.031*	
H11C	−0.0705	−0.4118	0.0823	0.031*	
H3	−0.141 (3)	−0.280 (4)	−0.102 (2)	0.050 (10)*	

### Atomic displacement parameters (Å^2^)

**Table:**

	*U*^11^	*U*^22^	*U*^33^	*U*^12^	*U*^13^	*U*^23^
Mn1	0.0121 (2)	0.0150 (3)	0.0109 (2)	0.0008 (2)	0.00264 (18)	0.0002 (2)
O1	0.0141 (8)	0.0196 (8)	0.0121 (7)	0.0013 (6)	0.0045 (6)	0.0006 (6)
O2	0.0180 (8)	0.0358 (10)	0.0161 (8)	0.0018 (7)	0.0040 (7)	0.0060 (7)
O3	0.0188 (8)	0.0180 (8)	0.0143 (8)	−0.0024 (7)	0.0007 (7)	0.0003 (7)
N1	0.0132 (9)	0.0113 (9)	0.0131 (9)	−0.0014 (7)	0.0035 (7)	−0.0022 (7)
C1	0.0121 (10)	0.0118 (11)	0.0168 (11)	−0.0023 (8)	0.0035 (9)	−0.0017 (9)
C2	0.0149 (11)	0.0158 (11)	0.0179 (11)	0.0003 (9)	0.0027 (9)	0.0044 (9)
C3	0.0150 (11)	0.0148 (11)	0.0248 (12)	0.0041 (9)	0.0032 (10)	0.0033 (10)
C4	0.0154 (11)	0.0113 (10)	0.0200 (12)	−0.0015 (8)	0.0049 (9)	−0.0031 (9)
C5	0.0141 (11)	0.0157 (11)	0.0265 (13)	0.0004 (9)	0.0078 (10)	−0.0037 (10)
C6	0.0197 (12)	0.0205 (12)	0.0224 (12)	−0.0037 (10)	0.0120 (10)	−0.0067 (10)
C7	0.0206 (12)	0.0200 (11)	0.0148 (11)	−0.0023 (9)	0.0053 (10)	−0.0010 (9)
C8	0.0144 (11)	0.0153 (11)	0.0158 (11)	0.0013 (8)	0.0037 (9)	−0.0026 (8)
C9	0.0142 (11)	0.0095 (11)	0.0167 (11)	−0.0022 (8)	0.0056 (9)	−0.0018 (9)
C10	0.0144 (11)	0.0147 (11)	0.0119 (11)	−0.0028 (9)	−0.0001 (9)	0.0003 (9)
C11	0.0228 (12)	0.0167 (12)	0.0223 (12)	−0.0006 (10)	0.0058 (10)	0.0002 (10)

### Geometric parameters (Å, °)

**Table:**

Mn1—O1	2.100 (2)	C4—C9	1.424 (3)
Mn1—O3	2.209 (2)	C4—C5	1.424 (3)
Mn1—N1	2.308 (2)	C5—C6	1.363 (3)
Mn1—O1^i^	2.100 (2)	C6—C7	1.406 (3)
Mn1—O3^i^	2.209 (2)	C7—C8	1.372 (3)
Mn1—N1^i^	2.308 (2)	C8—C9	1.420 (3)
O1—C10	1.271 (3)	C2—H2A	0.93
O2—C10	1.241 (3)	C3—H3A	0.93
O3—C11	1.436 (3)	C5—H5A	0.93
O3—H3	0.80 (3)	C6—H6A	0.93
N1—C1	1.325 (3)	C7—H7A	0.93
N1—C9	1.377 (3)	C8—H8A	0.93
C1—C10	1.529 (3)	C11—H11A	0.96
C1—C2	1.413 (3)	C11—H11B	0.96
C2—C3	1.367 (3)	C11—H11C	0.96
C3—C4	1.409 (3)		
			
O1—Mn1—O3	89.23 (7)	C3—C4—C5	123.9 (2)
O1—Mn1—N1	74.93 (7)	C4—C5—C6	120.9 (2)
O1—Mn1—O1^i^	180.00	C5—C6—C7	120.4 (2)
O1—Mn1—O3^i^	90.77 (7)	C6—C7—C8	121.1 (2)
O1—Mn1—N1^i^	105.07 (7)	C7—C8—C9	119.6 (2)
O3—Mn1—N1	88.01 (7)	N1—C9—C8	119.1 (2)
O1^i^—Mn1—O3	90.77 (7)	C4—C9—C8	119.8 (2)
O3—Mn1—O3^i^	180.00	N1—C9—C4	121.06 (19)
O3—Mn1—N1^i^	91.99 (7)	O2—C10—C1	118.6 (2)
O1^i^—Mn1—N1	105.07 (7)	O1—C10—O2	125.0 (2)
O3^i^—Mn1—N1	91.99 (7)	O1—C10—C1	116.33 (18)
N1—Mn1—N1^i^	180.00	C1—C2—H2A	121.00
O1^i^—Mn1—O3^i^	89.23 (7)	C3—C2—H2A	121.00
O1^i^—Mn1—N1^i^	74.93 (7)	C2—C3—H3A	120.00
O3^i^—Mn1—N1^i^	88.01 (7)	C4—C3—H3A	120.00
Mn1—O1—C10	120.67 (14)	C4—C5—H5A	120.00
Mn1—O3—C11	121.70 (13)	C6—C5—H5A	120.00
C11—O3—H3	105 (2)	C5—C6—H6A	120.00
Mn1—O3—H3	116 (2)	C7—C6—H6A	120.00
Mn1—N1—C9	129.59 (14)	C6—C7—H7A	119.00
C1—N1—C9	118.96 (19)	C8—C7—H7A	119.00
Mn1—N1—C1	111.36 (15)	C7—C8—H8A	120.00
C2—C1—C10	120.13 (19)	C9—C8—H8A	120.00
N1—C1—C2	123.2 (2)	O3—C11—H11A	109.00
N1—C1—C10	116.64 (19)	O3—C11—H11B	109.00
C1—C2—C3	118.6 (2)	O3—C11—H11C	109.00
C2—C3—C4	120.3 (2)	H11A—C11—H11B	109.00
C3—C4—C9	117.9 (2)	H11A—C11—H11C	109.00
C5—C4—C9	118.26 (19)	H11B—C11—H11C	110.00
			
O3—Mn1—O1—C10	89.82 (16)	C1—N1—C9—C4	−1.3 (3)
N1—Mn1—O1—C10	1.67 (15)	Mn1—N1—C9—C4	174.99 (15)
O3^i^—Mn1—O1—C10	−90.18 (16)	Mn1—N1—C9—C8	−5.6 (3)
N1^i^—Mn1—O1—C10	−178.33 (15)	C10—C1—C2—C3	−179.0 (2)
O1—Mn1—O3—C11	−149.62 (16)	C2—C1—C10—O1	176.7 (2)
N1—Mn1—O3—C11	−74.68 (16)	N1—C1—C10—O1	−1.5 (3)
O1^i^—Mn1—O3—C11	30.38 (16)	N1—C1—C10—O2	179.83 (19)
N1^i^—Mn1—O3—C11	105.32 (16)	N1—C1—C2—C3	−0.9 (3)
O1—Mn1—N1—C1	−2.35 (14)	C2—C1—C10—O2	−1.9 (3)
O1—Mn1—N1—C9	−178.82 (18)	C1—C2—C3—C4	0.1 (3)
O3—Mn1—N1—C1	−92.09 (14)	C2—C3—C4—C9	0.1 (3)
O3—Mn1—N1—C9	91.44 (17)	C2—C3—C4—C5	−179.2 (2)
O1^i^—Mn1—N1—C1	177.65 (14)	C3—C4—C9—N1	0.5 (3)
O1^i^—Mn1—N1—C9	1.18 (18)	C3—C4—C5—C6	178.7 (2)
O3^i^—Mn1—N1—C1	87.91 (14)	C9—C4—C5—C6	−0.6 (3)
O3^i^—Mn1—N1—C9	−88.56 (17)	C5—C4—C9—C8	0.4 (3)
Mn1—O1—C10—C1	−0.8 (3)	C3—C4—C9—C8	−178.9 (2)
Mn1—O1—C10—O2	177.74 (17)	C5—C4—C9—N1	179.8 (2)
Mn1—N1—C1—C2	−175.44 (17)	C4—C5—C6—C7	−0.1 (3)
C9—N1—C1—C2	1.5 (3)	C5—C6—C7—C8	1.1 (3)
C9—N1—C1—C10	179.63 (18)	C6—C7—C8—C9	−1.3 (3)
Mn1—N1—C1—C10	2.7 (2)	C7—C8—C9—N1	−178.9 (2)
C1—N1—C9—C8	178.2 (2)	C7—C8—C9—C4	0.5 (3)

Symmetry codes: (i) −*x*, −*y*, −*z*.

### Hydrogen-bond geometry (Å, °)

**Table:**

*D*—H···*A*	*D*—H	H···*A*	*D*···*A*	*D*—H···*A*
O3—H3···O2^ii^	0.80 (3)	1.83 (3)	2.623 (3)	172 (3)
C2—H2A···O1^iii^	0.93	2.58	3.411 (3)	148
C8—H8A···O1^i^	0.93	2.36	3.241 (3)	158

Symmetry codes: (ii) −*x*−1/2, *y*−1/2, −*z*−1/2; (iii) −*x*−1/2, *y*+1/2, −*z*−1/2; (i) −*x*, −*y*, −*z*.
